# Versatile and Highly Efficient MRI Simulation of Arbitrary Motion in KomaMRI

**DOI:** 10.1002/mrm.70145

**Published:** 2025-10-27

**Authors:** Pablo Villacorta‐Aylagas, Carlos Andrés Castillo‐Passi, Ryan Anders Kierulf, Rosa María Menchón‐Lara, Justino R. Rodríguez‐Galván, José Benito Sierra‐Pallares, Pablo Irarrazaval, Carlos Alberola‐López

**Affiliations:** ^1^ Laboratorio de Procesado de Imagen Universidad de Valladolid Valladolid Spain; ^2^ School of Biomedical Engineering and Imaging Sciences Kings College London UK; ^3^ Institute for Biological and Medical Engineering Pontificia Universidad Católica de Chile Santiago Chile; ^4^ Millennium Institute for Intelligent Healthcare Engineering (iHEALTH) Pontificia Universidad Católica de Chile Santiago Chile; ^5^ Department of Radiology Stanford University Stanford California USA; ^6^ School of Computer, Data & Information Sciences University of Wisconsin–Madison Madison Wisconsin USA; ^7^ Universidad Politécnica de Cartagena Región de Murcia Spain; ^8^ Department of Energy Engineering and Fluid Mechanics & ITAP Universidad de Valladolid Valladolid Spain; ^9^ Instituto de Investigación Biosanitaria de Valladolid (IBioVall) Valladolid Spain

**Keywords:** motion, MRI simulation, open‐source, performance

## Abstract

**Purpose:**

To extend the KomaMRI simulator with motion capabilities that enable the simulation of both simple, parametrizable motion patterns and arbitrarily complex spin trajectories. Additionally, we introduce a novel file format for storing and sharing dynamic digital phantoms.

**Methods:**

The existing Phantom structure in KomaMRI has been extended with a new motion field, which preserves support for static phantoms and introduces the capability to define dynamic models, composed of either a single or an arbitrary set of elementary motions. Each motion entry is composed of an *action*, a *time curve*, and a *spin span*, allowing for modular definition and full parameter independence across motion components. An HDF5‐based phantom file format has been defined, along with two I/O functions within the simulator. Motion‐related MRI experiments have been conducted for both illustrative and comparative evaluation.

**Results:**

Illustrative experiments reveal motion‐related effects commonly observed in real MRI, such as time of flight and phase contrast. Comparative evaluations show strong qualitative and quantitative agreement with results reported by other simulation tools and with at least a three‐fold reduction in computation time.

**Conclusions:**

The proposed extension adds flexible motion modeling and simulation capabilities to KomaMRI. The proposed new functions allow for accurate and high temporal and spatial resolution, only limited by computational cost.

## 1 Introduction

MRI simulation is a valuable tool for sequence development, analysis of isolated phenomena, and synthesis of images for further usage (e.g., training of learning models or creation of signal dictionaries) [1]. It also serves as a powerful resource for education and training [2]. In recent years, a number of physics‐based simulators have been released, including open‐source tools such as JEMRIS [3], MRiLab [4, 5], KomaMRI [1], and CMRsim [6]. Proprietary options have also been reported, such as BlochSolver [7] or Corsmed, which is an evolution of MRISIMUL [8, 9] and coreMRI [10]. We now focus on JEMRIS and CMRsim due to their open‐source nature, active maintenance, support for complex motion, and closeness to KomaMRI, the extension of which is the purpose of our paper. Occasionally, other simulators will also be referred to. JEMRIS (Jülich Extensible MRI Simulator) [3] is the most popular open source MRI simulator. It was developed in C++ and includes three MATLAB GUIs for interactive sequence design, multiple coil configuration, and simulation execution. Additionally, it provides Pulseq support. Moreover, it uses CPU multithreading via the MPI library; however, GPUs are currently unsupported. JEMRIS first provided support for rigid motion; then, two extensions have provided additional capabilities, namely, the *TrajectoryFlow* class [11, 12] that enables the description of flow phenomena [11] and the non‐rigid deformable phantoms [13], for cardiac MR (CMR) or respiration. Overall, JEMRIS provides realistic simulations. On the downside, configuring motion is often complex and time‐consuming, and simulation times are quite long [12, 13]. CMRsim [6] is a newly released framework that enables the simulation of CMR. It was designed to simplify the task of defining complex motion; the framework also provides modularity for designing comparative simulation experiments. Furthermore, CMRsim promotes reproducibility through versioned and documented open‐source software, and it is readily scalable to support detailed digital phantoms. In addition, it supports Pulseq and includes CMRseq [14], a sequence design tool. As for advantages, CMRsim includes simple installation, open‐source nature, multiple coil support, and easy definition of complex movements. As for limitations, CMRsim is written in Python, hence speed—even with TensorFlow CPU/GPU acceleration—is limited, and on‐the‐fly trajectory calculations for flow data further reduce performance. KomaMRI [1] is a Julia package which inherits the strengths of this programming language [15], runs *as is* in all major operating systems and provides a GUI and vendor‐agnostic GPU‐accelerated simulations giving rise to computationally efficient simulations. KomaMRI is compatible with key MRI standards, such as Pulseq [16] and ISMRMRD [17]. The limitations of this simulator have been described elsewhere [1] or are reported as known issues.^1^, ^2^ Its main limitation is the fact that motion must be defined using analytical expressions, which makes modeling simple movements unnecessarily difficult and restricts the definition of complex motion patterns. In this paper, we overcome this limitation while maintaining its computational efficiency. We show that our extension reproduces more efficiently the experiments reported by other MRI simulators. A common limitation of current MRI simulators is the absence of a standardized file format for digital phantoms, which hinders data sharing and reproducibility. In contrast, sequence files already follow the widely adopted Pulseq [16] format. Here, we also propose a similar standard for digital phantoms.

## 2 Methods

The digital phantom of KomaMRI is defined by the Phantom structure (see Supporting Information Code S1a). Initial positions (x, y, z), contrast‐related (T1, T2, T2s, ρ ) information, and off‐resonance effects (Δω) associated with each spin, are stored as vectors. Three functions of time (namely, ux, uy, and uz) are expected to analytically define spin displacements. Replacing these functions with a more versatile solution is our purpose.

### 2.1 Design Considerations

We aim to provide support for the following functionality:

1. Mix‐and‐match combinations of multiple motions, either simultaneous, sequential, or overlapping, including simple (rotations, translations) and complex physiological patterns (breathing, heartbeat).
2. Pseudo‐periodic motion, such as irregular respiratory motion or cardiac arrhythmia.
3. Arbitrary spin trajectories, for flow or diffusion.
4. Spin‐specific motion definitions, to specify affected/unaffected spins by the motion model.
5. Arbitrary temporal transitions between the initial and final states of motion.

### 2.2 Proposed Model

#### 2.2.1 Overview

**FIGURE 1:**
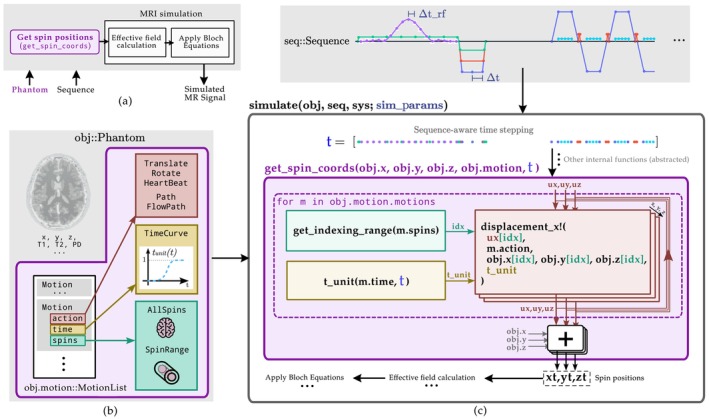
Code overview of the implemented model. The main contributions presented in this paper are highlighted in purple. (a) Conceptual MRI simulation pipeline, where the Phantom and sequence are shown as input arguments to the pipeline (gray block), and where the computation of spin positions is performed prior to the actual MRI simulation. (b) Extension of the Phantom structure to include a new motion field, which is, in its most general form, a list of Motion instances, each defined by an action, a time curve, and a spin span. (c) Operation of the get_spin_coords function within the simulate workflow. For each motion instance defined in the Phantom, displacements are computed based on its time curve and affected spin range. These displacements are added to the initial spin positions to generate the xt, yt, and zt matrices, which are then used to compute the effective magnetic field and solve the Bloch equations.

Figure 1a shows a diagram of the simulation pipeline, with our contributions highlighted in purple. These contributions include the modification of the Phantom structure and the introduction of the get_spin_coords function. The former, illustrated in Figure 1b, consists of the addition of the motion field to the Phantom structure.^3^ In its general form, this field consists of a list of Motion instances, each defined as a combination of three entities: An action, a time curve, and a spin span. Simpler cases—such as a single Motion instance or a NoMotion instance (for static phantoms)—are also supported. The latter, detailed in Figure 1c, is a function that computes the position of each spin at the time points required by the sequence. This is done by computing the displacements from the motionfield at each time instant and adding them to the initial spin positions. The function is called internally by the core routines run_spin_excitation! and run_spin_precession!, which store the resulting coordinates in the xt, yt, and zt matrices, subsequently used in the simulation. We use an example to provide an overview of our proposal. The example consists of a simple translation of an object, as shown in Code 1: 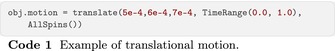


As indicated in Figure 1b, Translate is one of the possible actions for the motion. The first three arguments are the three scalars that define the displacement in x, y, and z coordinates. Then, the TimeRange method indicates that this translation should start at time instant zero and be completed at time instant 1 (in seconds). Finally, the method AllSpins causes the motion to affect all the spins. Assume now that we want to undo the translation, moving the object back to its original position at twice the speed. In addition, only a fraction of the spins should move. The former is achieved by the TimeCurve concept and the latter by the SpinRange method, as shown in Code 2: 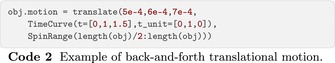


The TimeCurve entity is analogous to the so‐called *animation curves* used in animation software. It defines a mapping between variables t and $t_{unit}$ (Figure 1b), where t is a sequence‐aware vector of time points at which calculations are performed, and $t_{unit}$ is a normalized variable ranging from 0 (start of the trajectory) to 1 (end of the trajectory). The mapping is specified via a piecewise linear interpolation between the nodes provided as input (t and t_unit in Code 2). For our example, this TimeCurve consists of two linear segments; the first maps the time interval (0,1) s to a full forward translation (t_unit from 0 to 1), and the second maps the time interval (1,1.5) s to a full backward translation (t_unit from 1 to 0). This implies that the displacements are applied progressively over time to the original spin positions to reflect this translation. Finally, SpinRange expects an enumeration of spins to be affected by the motion.

#### 2.2.2 Actions

The five actions that can be currently executed are Translate, Rotate, HeartBeat, Path, and FlowPath. The first three fall under the category of *SimpleActions* while the last two belong to *ArbitraryActions*. They are treated differently, as explained below. First, we will describe the actions in isolation, then within a MotionList.

##### 2.2.2.1 Simple Actions

Simple actions are characterized by an explicit dependence on the variable $t_{unit}$, which defines the motion progression over time and can be mathematically expressed as

(1) $$t_{unit}(t;\Theta ),$$

with Θ a parameter vector that comprises the nodes that define the mapping. The type of motions in this category are, as indicated in Figure 1b, translation, rotation, and heartbeat. Translations and rotations are straightforward to define.^4^ The heartbeat is a motion defined in cylindrical coordinates depending on three parameters: circumferential_strain, radial_strain, and longitudinal_strain. Let (r, θ, z) be the coordinates of spin position, and let r represent the vector consisting of the r values of the spins within the heart. Then, the displacements are defined as:

$$\begin{array}{ll} \Delta_{\text{circ}} & =\text{circumferential\_strain}\cdot \max(r)\\ \Delta_{\text{rad}} & =-\text{radial\_strain}\cdot (\max(r)-r)\\ \Delta_{r}(t) & =(\Delta_{\text{circumferential}}+\Delta_{\text{radial}})\cdot t_{unit}(t;\Theta )\\ u_{x}(t) & =\Delta_{r}(t)\cdot \cos(\theta )\\ u_{y}(t) & =\Delta_{r}(t)\cdot \sin(\theta )\\ u_{z}(t) & =\text{longitudinal\_strain}\cdot z\cdot t_{unit}(t;\Theta ) \end{array}$$

##### 2.2.2.2 Arbitrary Actions

Arbitrary actions enable the user to directly specify a trajectory for each spin. These trajectories consist of three displacement matrices with as many rows as spins and each row containing a series of displacements at equispaced points in time. However, both the velocity and the forward/backward ordering in which the trajectories are traversed still depend on the defined TimeCurve. Two types of arbitrary actions have been defined: Path and FlowPath. Both share the aforementioned matrices, but FlowPath has an additional field, spin_reset, which enables flow support. In this case, spins may leave the volume in which the phantom is defined, and new unexcited (*fresh*) spins may enter. Hence, spins that leave are reinjected back into the phantom, and their magnetic state is reset to an unexcited state. Overall, the spin number along the simulation remains constant. spin_reset is implemented as a binary matrix with a 1 entry in the time instant in which the spin leaves the volume. Note that both the spin trajectories and the spin_reset matrix must be provided by the user when designing the phantom. These data can be generated using third‐party software such as our in‐house VTK‐m solver.^5^

##### 2.2.2.3 Actions Within a MotionList

In the case that a MotionList is used—i.e., two or more motions are combined—actions are ordered according to their temporal configuration and executed according to their dependencies: Rotations and deformations depend on the updated spin positions and must therefore be executed sequentially (hence the feedback loop from the displacement! function in Figure 1c); translations, on the other hand, can be computed and added directly to the original coordinates due to their independence from updated positions. This guarantees that rotations and deformations are resolved first and the rotation center—whether user‐defined or, by default, the center of mass—is consistently referenced. This approach enables the definition of complex yet physically consistent motion patterns. For instance, realistic rigid head motion, as demonstrated in an illustrative example,^6^ can be accurately reproduced by combining sequential and overlapping actions within a MotionList.

#### 2.2.3 Time Curve

Recall that TimeCurve is the entity used to control the temporal behavior of the trajectory through Equation (1). Moreover, it has two additional parameters, namely, periodic and periods. periodic is Boolean and indicates whether Equation (1) should be periodically extended beyond the time interval used for its definition. periods is a vector of positive real components that indicate the number of interval extensions that should be done and the time length of each extension. This enables pseudo‐periodic extensions with time warping. Notice that TimeRange in Code 1 defines a TimeCurve with an identity mapping. Additional examples are:

##### 2.2.3.1 Simple Non‐Periodic Motion


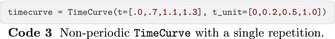


**FIGURE 2:**
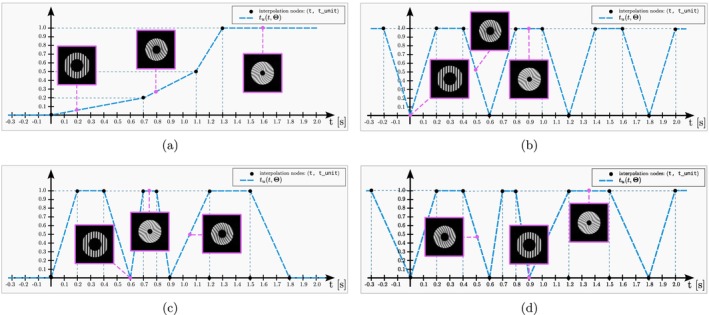
Four examples of TimeCurve instances. The pink‐bordered frames show the state of the phantom at each time point. (a) A non‐periodic curve with flat extrapolation applied outside the time curve limits. (b) A periodic curve with a single repetition. (c) A non‐periodic curve with three repetitions of variable durations. (d) A periodic extension of (c).

The arguments t and t_unit define Θ in Equation (1) so that the resulting curve is the one shown in Figure 2a. The curve indicates that at time instant t=1.1 s, 50% of the motion is carried out, and after t=1.3 s the object remains still.

##### 2.2.3.2 Simple Periodic Motion


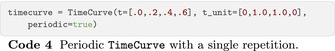


Code 4 defines a periodic trajectory of period 0.6 (see Figure 2b). The object completes its trajectory in 0.2 s, remains still for 0.2 s, and then returns to its original position (again, in 0.2 s). The process is repeated indefinitely.

##### 2.2.3.3 Composed Non‐Periodic Motion


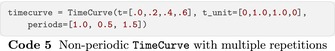


Code 5 defines a pseudo‐periodic trajectory (see Figure 2c). The second pseudo‐period lasts half as long as the first, whereas the third is 50% longer than the first.

##### 2.2.3.4 Composed Periodic Motion


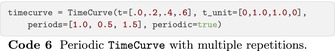


In this case (see Figure 2d), the situation depicted in Figure 2c is periodically extended.

### 2.3 Phantom File Format

To address the aforementioned lack of a standardized format for digital phantoms, we propose a new file format, .phantom, designed to store comprehensive digital phantom data for MRI simulations. The format is intended to accommodate a wide range of essential information, including initial spin positions, motion trajectories, relaxation properties ($T_{1}$, $T_{2}$, PD), off‐resonance effects, and relevant metadata. Given the complexity and size of these data, the format is based on HDF5, a widely supported standard across scientific computing environments. The main objective of .phantom is to promote interoperability and reproducibility across different MRI simulation environments by providing a standardized, extensible, and efficient solution to encode, store, and share digital phantoms. This format is currently fully compatible with KomaMRI, which now includes built‐in I/O functions for handling .phantom files: read_phantom and write_phantom. A complete description of the proposed file format, including its motivation, an overview of the HDF5 standard, and detailed explanations of all included data fields, can be found at Supporting Information S2 and at this location: https://juliahealth.org/KomaMRI.jl/phantom‐format/.

### 2.4 Experiments

Two types of experiments were conducted in KomaMRI v0.9.2. First, an illustrative experimentation, which highlights the model's flexibility and ease of use by replicating two realistic motion‐related MRI acquisitions. Sections 2.4.1 and 2.4.2 describe this type of experiment. Second, a comparative experimentation with established MRI simulators, to qualitatively and quantitatively test model performance. This second group comprises the experiments described in Sections 2.4.3, 2.4.4, 2.4.5. All experimental settings—including phantom characteristics, pulse sequence parameters, and result details—are provided in Supporting Information S3. Unless otherwise specified, simulations were run on a desktop computer (NVIDIA Quadro RTX 4000, 8 GB). Some experiments were instead executed on a server (4 × NVIDIA RTX A5000 GPUs, 24 GB). These two configurations will hereafter be referred to as *desktop* and *server*, respectively (see Table S3a in Supporting Information).

#### 2.4.1 Time‐of‐Flight on a User‐Defined Flow Phantom

In this experiment, the phantom is modeled as a vertical cylindrical tube aligned with the z axis, with a continuous uniform flow in the positive z direction. This is implemented using a periodic TimeCurve and a FlowPath action to reinject particles reaching the upper boundary. The flow velocity is set so that the cylinder length is traversed in one period. A cine sequence based on b‐SSFP is used for two separate simulations in different planes. Full details of both the phantom and sequence are provided in Table S3b. A graphical representation of the phantom and acquisition planes is shown in the Results section.

#### 2.4.2 Phase Contrast on an Aorta Model

**FIGURE 3:**
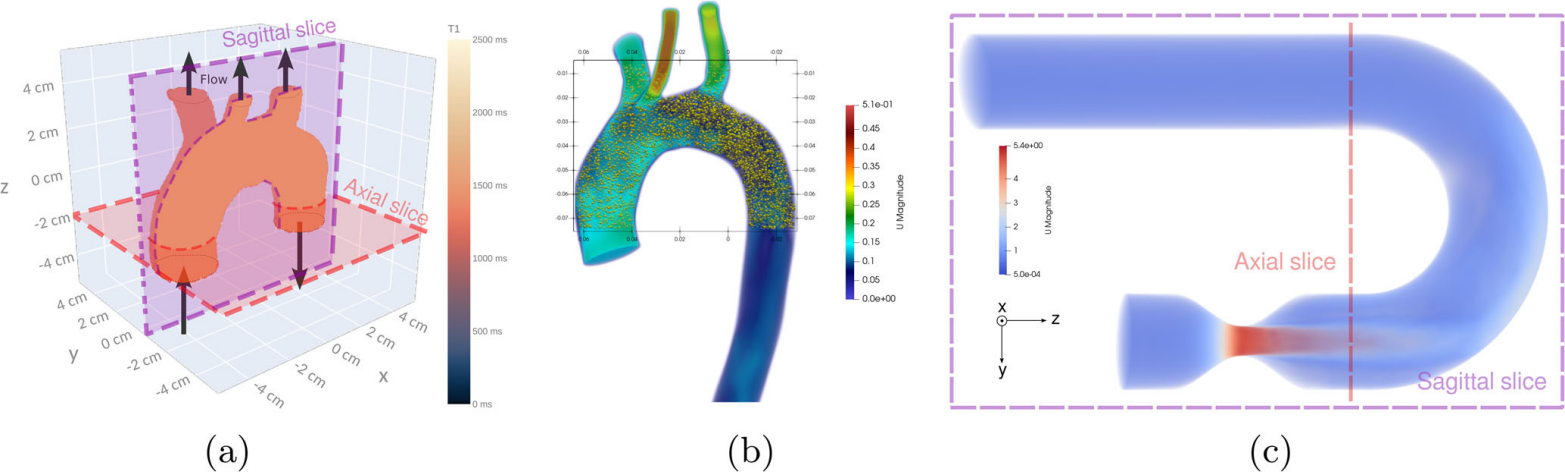
(a) Aorta phantom composed of two million spins. (b) FOV of the synthetic aorta and initial positions of a subset of spins. (c) Volume rendering of a stenotic U‐bend velocity field, visualized in Paraview [21]. Dashed purple and red lines indicate the orientations of the sagittal and axial planes, respectively.

The phantom used for this experiment is shown in Figure 3a. The anatomical information is extracted from the *Vascular Model Repository* ^7^ and the flow field over this anatomy was computed using OpenFOAM [18]. We have randomly seeded two million spins throughout the volume of interest (Figure 3b), and their trajectories have been calculated using a particle trajectory solver developed in‐house with VTK‐m [19, 20], that takes as input the velocity field files in VTK legacy format and a seed file that contains the initial positions of these particles. Trajectories are resolved between t = 0 and t = 1 s, with a time step of 0.5 ms. These trajectories were sampled every 10 ms, providing a reasonable time resolution that generates a manageable file size of approximately 3 GB. As a result, we obtain a total of 100 positions per spin. Anytime a spin leaves the aorta, it is reseeded and matrix spin_reset is updated accordingly. The pulse sequence used is a multi‐shot GRE‐EPI. Two slices have been simulated: Axial (red square in Figure 3a) and sagittal (purple square). Additional details of both the phantom and the sequence are provided in Table S3c.

#### 2.4.3 Validation of the Bloch Solver Under Flow Conditions

##### 2.4.3.1 Qualitative Comparison

The validation experiment originally proposed in Reference [22] and later conducted in References [23, 24] has been replicated. The phantom designed for this experiment consists of a 1D segment along the z direction, populated with an evenly‐spaced set of spins. The sequence, described in Reference [22], has been simulated and compared with [23] (see Table S3d). The magnetization has been recorded at the end of the rewinder gradient, as marked by the arrow in Figure 7b from Reference [24].

##### 2.4.3.2 Quantitative Comparison

Additionally, a quantitative comparison has been performed using the DifferentialEquations.jl [25] suite, specifically, the package OrdinaryDiffEq.jl. To ensure a highly accurate reference solution, the fifth‐order Runge–Kutta method was employed. A total of 50 spins were simulated, and various time step sizes—ranging from $10^{-5}$ to $10^{-7}$ s—were tested in KomaMRI.

#### 2.4.4 Myocardial Tagging on a User‐Defined Phantom

**FIGURE 4:**
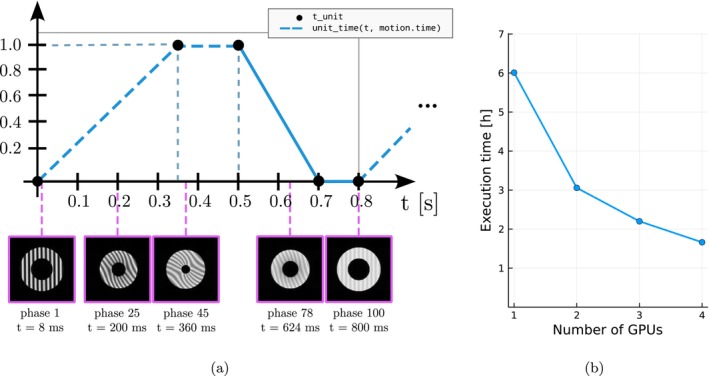
(a) Graphical representation of Code 7, where systole is shown as a dashed line and diastole as a solid line (following Figure 2 of [9]). Below the graph, five frames from the 100 acquired during a 2D tagged cine simulation are displayed at their corresponding time points. These results, obtained in KomaMRI, are virtually identical to those reported in [9]. (b) Evolution of execution times in KomaMRI as the number of GPUs increases. The graph shows a non‐linear decrease due to both Amdahl's Law [27] and to the subdivision of the phantom, composed of 7 million spins, into fragments that fit into the GPU. This required certain parts of the simulation to be performed sequentially rather than in parallel.

The work of Tecelao et al. [26] presents a myocardium deformation model, which was later used by Xanthis et al. [9] in a cardiac MRI simulation experiment. This experiment has been replicated in KomaMRI (see Table S3e). For the deformation model, a Path action was used. For the temporal behavior, Code 7 shows the instruction used and Figure 4a illustrates it graphically; this leads to a motion pattern identical to the one shown in Figure 2 of [9]. Finally, the pulse sequence used in Reference [9] has also been replicated. 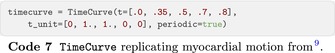 This experiment has been conducted on *server* to enable comparison with [9] and to study execution times as a function of the number of GPUs used. The phantom has been partitioned into as many parts as available GPUs. Each part has been further subdivided into smaller units to prevent memory overflow.

#### 2.4.5 Phase Contrast Comparisons

##### 2.4.5.1 Comparison With CMRsim

We have replicated the flow experiment described in Reference [6], which involves seven velocity‐encoded spoiled GE acquisitions with varying directions and $V_{\text{ENC}}$ values, applied to a stenotic U‐bend phantom (see Figure 3c). The velocity field file was obtained from the CMRsim repository,^8^ and the sequence details are described in Reference [6]. As for CMRSim, the temporal grid size was set to 10 μs. The target density for both initial seeding and reseeding was 5 spins per mm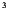, resulting in a total of 1.5 million particles [6]. The so‐called *TurbulentTrajectory* CMRsim module is used, and spin trajectories are calculated on‐the‐fly together with the simulation. Our method, on the other hand, uses the VTK‐m solver to pre‐calculate these trajectories and store them in a .phantom file. The 1.5 million spins are uniformly distributed at random positions throughout the volume. As a second experiment, we attempt to compare an axial PC‐MRI slice (in the x–y plane, Figure 3c, red dashed line) of the U‐bend, acquired with KomaMRI, directly against the non‐turbulent ground truth CFD data. In this case, the phantom was populated with 5 million spins, with no turbulence added to their trajectories. Details of both experiments are summarized in Table S3f.

##### 2.4.5.2 Comparison With JEMRIS

This experiment replicates the phase contrast acquisition from Reference [12]. The digital phantom consists of two parallel tubes with opposite flow directions, one of which is surrounded by static tissue (see Figure 10). See [12] for details of the pulse sequence. For JEMRIS, spin trajectories were provided to the simulator via the *FlowTrajectories* input field as an ASCII file. The pulse sequence, on the other hand, is a .xml file, which is automatically downloaded to the JEMRIS folder during its installation. As for KomaMRI, the ASCII spin trajectories were interpolated at 400 evenly spaced time instants between 0 and 7 s, and then integrated into a .phantom file along with other parameters such as $T_{1}$ and $T_{2}$. This resulted in a phantom file of approximately 7 GB. Some noise was added, following [12], although its intensity was fine‐tuned since [12] does not specify its parameters. The pulse sequence was generated using the KomaMRI API; we checked that importing the JEMRIS .seq file provided the same sequence. See Table S3g for further details.

## 3 Results

### 3.1 Illustrative Evaluation

#### 3.1.1 Time of Flight on a User‐Defined Flow Phantom

**FIGURE 5:**
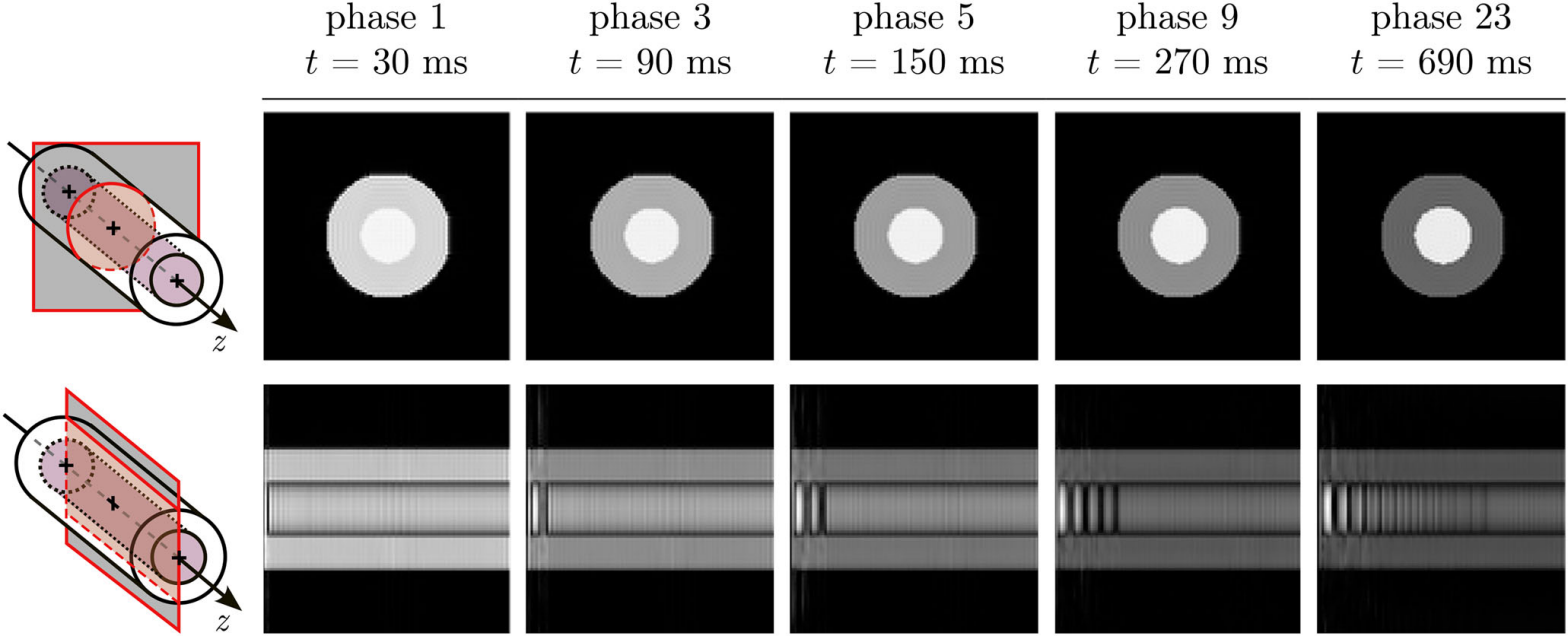
Five of the 30 acquired phases, which reflect the steady state reached by the wall spins. These spins emit less signal compared to the fresh spins injected through the lower entry of the phantom, which correspond to the left side of the frames in the second row. The flow velocity is 4 cm/s. The phantom consists of 466 722 spins, and both simulations were completed in a total of 24 min.

TOF effects encountered in 2D‐GE sequences are noticeable and particularly evident in the axial frames shown in the first row of Figure 5. In these images, static spins reach a steady state and look darker, while flowing spins are brighter. Similar considerations can be made for the longitudinal frames (second row), in which newly entered spins look brighter while those that remain longer in the plane reach a steady state and provide less signal. Each of the two acquisitions—axial and longitudinal—took approximately 12 min.

#### 3.1.2 Phase Contrast on an Aorta Model

**FIGURE 6:**
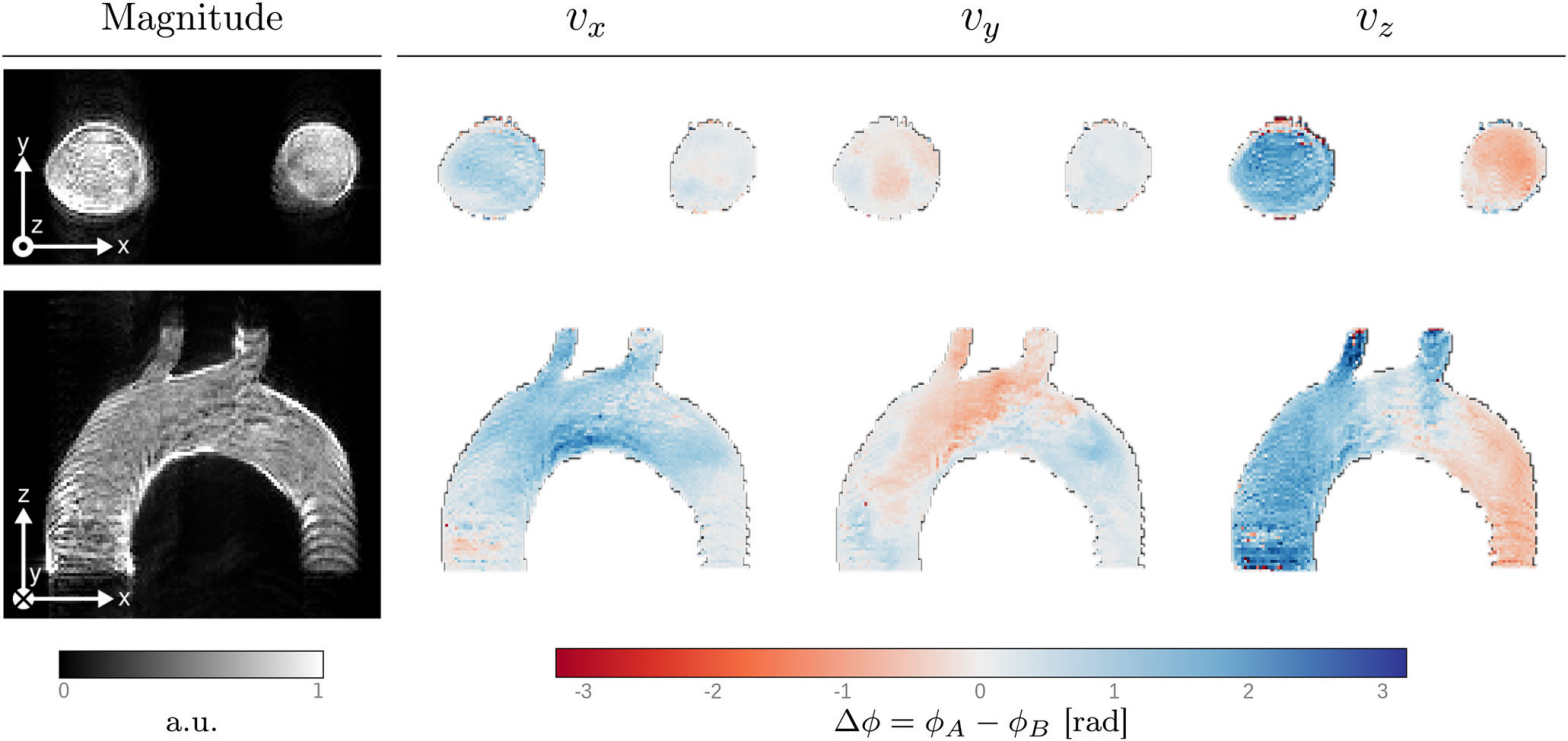
Results of the PC‐MRI experiment performed on a flow aorta phantom composed of two million spins. In the magnitude image, especially in the sagittal slice, the increased brightness and contrast at the edges of the aorta indicate the accumulation of spins along the vessel walls. In the phase images, the color represents the flow direction in each axis, while the intensity reflects its magnitude relative to $V_{\text{ENC}}$ of 50 cm/s (i.e., with respect to Δϕ=π). Accordingly, the $v_{z}$ component exhibits the highest values, while the $v_{y}$ component shows the smallest values. Total simulation time was 26 min.

Figure 6 shows the results, with axial images in the first row and sagittal images in the second. The first column shows the magnitude images (obtained from the modulus of the x‐component). The next three columns show the phase difference images Δϕ that result from encoding the flow velocity in the x, y, and z directions with toggled bipolar gradients. To avoid clutter, a mask has been applied to these phase images to exclude pixels where the magnitude image does not exceed a certain intensity threshold. In the magnitude images, the increased brightness and contrast at the vessel edges, particularly in the sagittal slice, highlight the accumulation of spins along the aortic walls. In the phase images, the color indicates the flow direction for each velocity component, while the intensity reflects the relative magnitude of the flow with respect to the $V_{\text{ENC}}$ of 50 cm/s; $v_{z}$ exhibits the largest values, whereas $v_{y}$ shows the smallest, consistent with the flow geometry of the phantom. A total of 12 images has been simulated (6 per slice, 2 per velocity component) to estimate the velocity vector. Each of these simulations took 2 min and 10 s.

### 3.2 Comparative Evaluation

#### 3.2.1 Validation of the Bloch Solver Under Flow Conditions

##### 3.2.1.1 Qualitative Comparison

**FIGURE 7:**
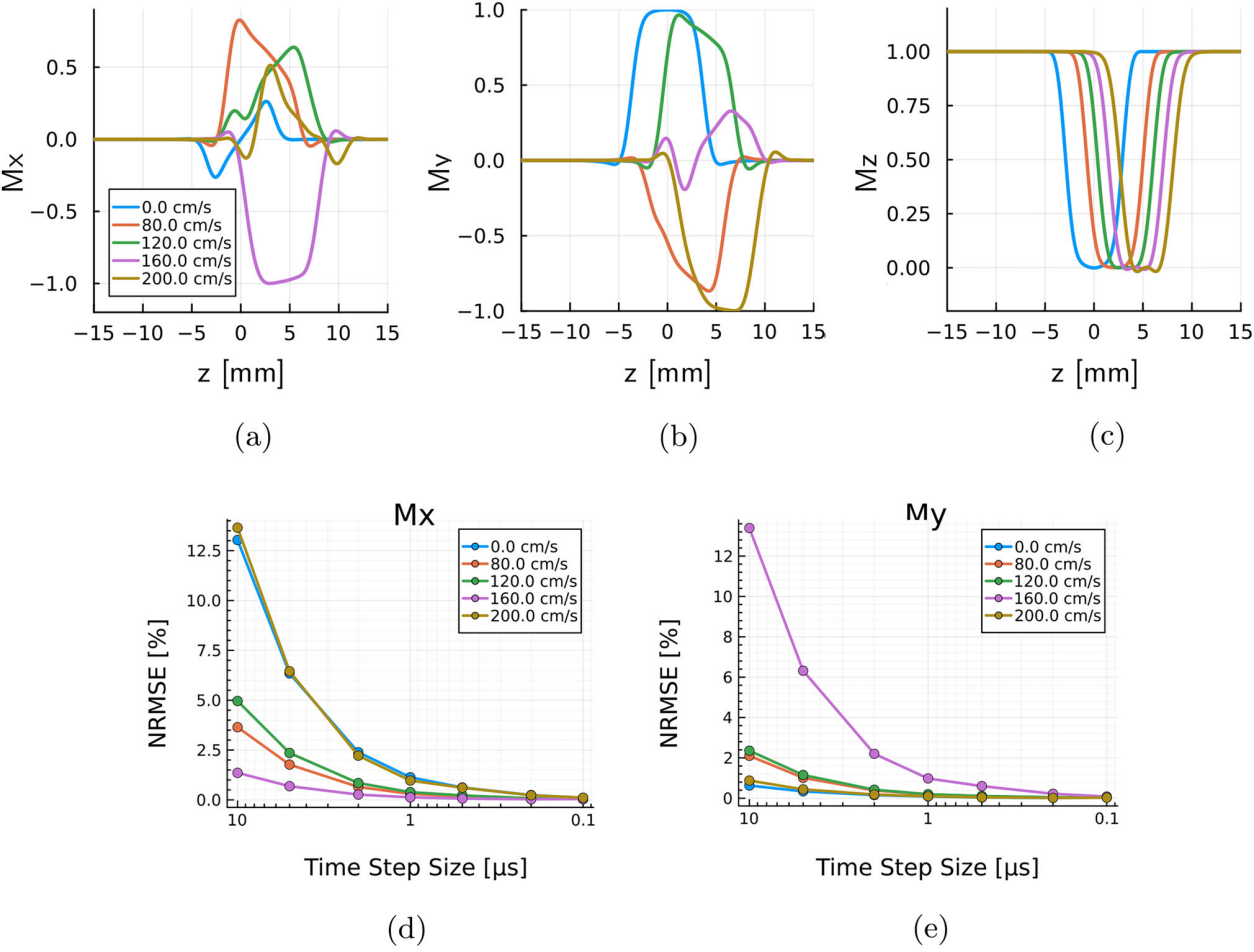
(a) Graphical representation of Code 7, where systole is shown as a dashed line and diastole as a solid line (following Figure 2 of [9]). Below the graph, five frames from the 100 acquired during a 2D tagged cine simulation are displayed at their corresponding time points. These results, obtained in KomaMRI, are virtually identical to those reported in [9]. (b) Evolution of execution times in KomaMRI as the number of GPUs increases. The graph shows a non‐linear decrease due to both Amdahl's Law [27] and to the subdivision of the phantom, composed of 7 million spins, into fragments that fit into the GPU. This required certain parts of the simulation to be performed sequentially rather than in parallel.

This experiment generated three magnetization profiles ($M_{x}$, $M_{y}$, and $M_{z}$) in the z‐direction for input velocities ranging from 0–200 cm/s. Figure 7a–c shows the magnetization profiles obtained using KomaMRI. These results exhibit an excellent agreement with those reported in Reference [23].

##### 3.2.1.2 Quantitative Comparison

Figure 7d,e shows the behavior of NRMSE with the step sizes and velocity sets. Although certain combinations—$M_{x}$ for v = [0, 200] cm/s and in $M_{y}$ for v = 160 cm/s—yield higher errors, they all decrease as the time step is reduced, reaching an acceptable value below 1% for Δt≃1μs. The minimum error of approximately 0.012% is obtained for Δt=0.1μs. Regarding $M_{z}$, the error scale is below 0.1% throughout the simulation, so it has been omitted.

#### 3.2.2 Myocardial Tagging on a User‐Defined Phantom

Figure 4a reveals the evolution of the contractility of the heart model in five of the SPAMM frames simulated. Visual similarity with [9] is noticeable, including contrast reduction due to $T_{1}$ relaxation. Figure 4b shows the execution times obtained using between 1 and 4 GPUs. Execution times decrease as the number of GPUs increases, although not linearly [27]. In every case, time is substantially shorter than the approximately 7 h reported by Xanthis et al. [9] with four GPUs.

#### 3.2.3 Phase Contrast

##### 3.2.3.1 Comparison With CMRsim

**FIGURE 8:**
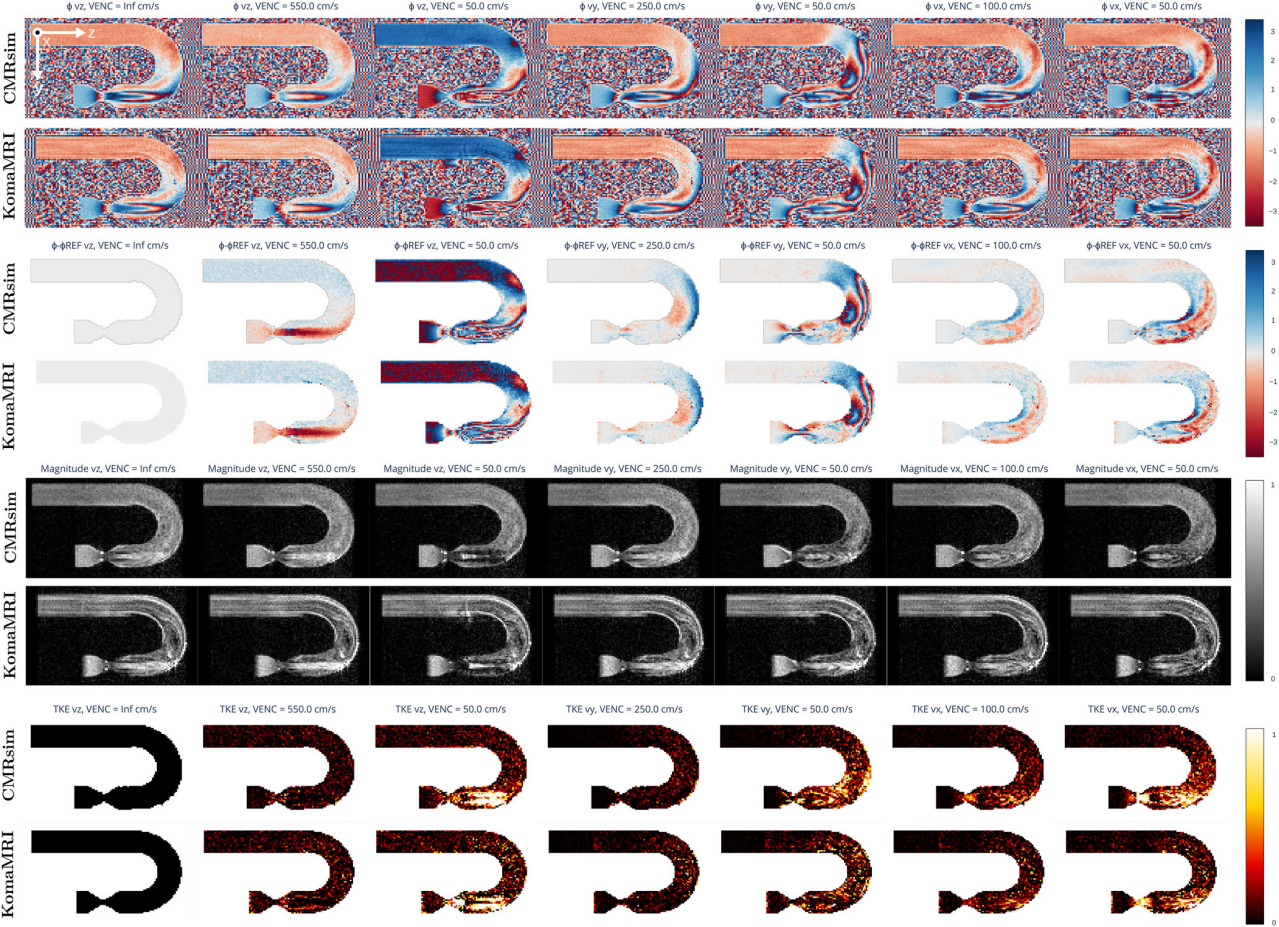
Simulation results for the PC‐MRI experiment of turbulent flow downstream of a stenotic U‐bend. From top to bottom, the images of phase, phase difference, magnitude, and TKE are shown for different $V_{\text{ENC}}$ and directions. The odd‐numbered rows correspond to the experiment conducted in CMRsim, while the even‐numbered rows correspond to the experiment replicated in KomaMRI. MRI simulation time in KomaMRI was 9 min, excluding the 39‐minute particle trajectory computation, which is required only once. In contrast, CMRsim required 53 min for both trajectory generation and MRI simulation, as these steps are not separable and must be performed in every new simulation.

**FIGURE 9:**
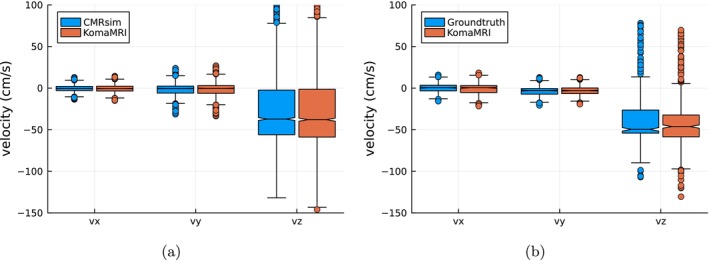
Boxplots of velocities obtained from (a) sagittal slices using both CMRsim and KomaMRI and (b) axial slices using the CFD actual calculations (referred to as GroundTruth) and KomaMRI. Both abscissae axes indicate the velocity component for each case.

Figure 8 shows the results for the sagittal slice (CMRsim odd‐numbered rows, KomaMRI even‐numbered rows). From top to bottom, we show the images of magnitude, phase, phase difference with respect to the reference, and the turbulent kinetic energy (TKE) coefficient [6]. Figure 9 shows a quantitative comparison of the velocity components of both CMRsim and KomaMRI. The boxplots show that velocity in the x‐component is essentially null. More relevant are the velocities in the two other components, which show strong agreement between simulators. Indeed, the Mann–Whitney U test [28] posits no shift between both distributions (*p*‐value > 0.35 for the three velocity components). The total duration of the CMRsim experiment was 53 min. This includes a single execution of the trajectory advection and reseeding steps, which were then applied in parallel to the seven $V_{\text{ENC}}$ acquisitions. In contrast, with our approach, the trajectory solver took 39 min, and the total MRI simulation time for the seven $V_{\text{ENC}}$ acquisitions was 9 min. Overall, 48 min on the same hardware. As for the axial slice, boxplots of the velocity components are shown in Figure 9b. Similar considerations as for the sagittal case apply; the Mann–Whitney U test [28] in the three components provides *p*‐values above 0.13, so, once again, distributions cannot be considered statistically shifted with respect to each other.

##### 3.2.3.2 Comparison With JEMRIS

**FIGURE 10:**
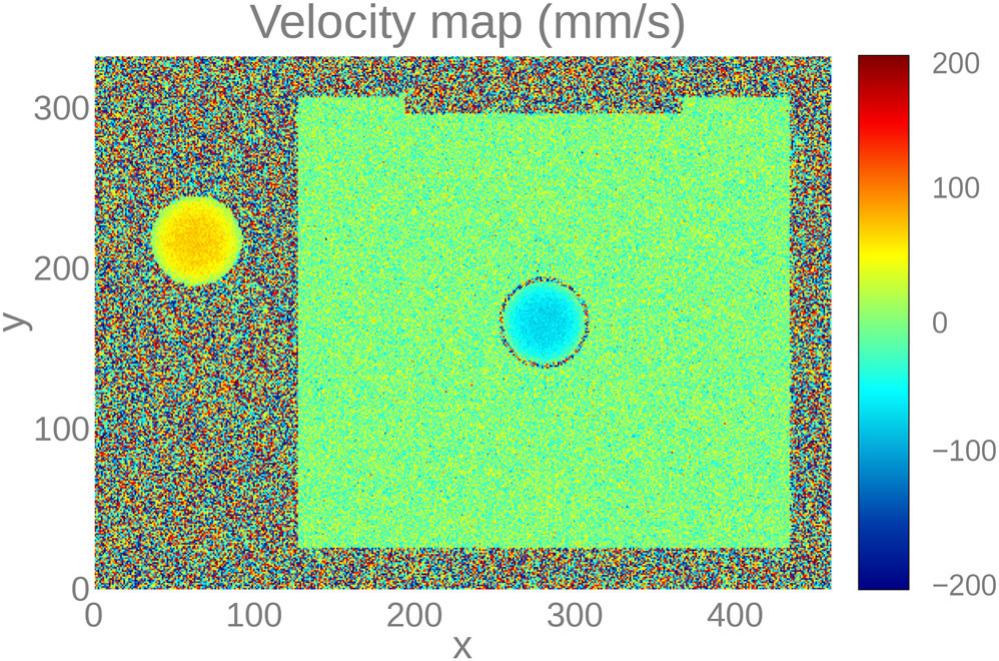
PC‐MRI results of the comparative experiment with JEMRIS. Phase contrast velocimetry conducted in KomaMRI over a digital phantom with flow data integrated from Poiseuille's law. The result is essentially indistinguishable from those in [12].

Each of the two $V_{\text{ENC}}$ simulations in JEMRIS required 134 h. In contrast, the corresponding $V_{\text{ENC}}$ acquisitions in KomaMRI required 21 min, leading to the result shown in Figure 10 and thus demonstrating good qualitative agreement with the experiment by Fortin et al. [12]. A quantitative comparison of velocity values is provided in Table S3h.

## 4 Discussion

Our proposal produced results consistent with theoretical expectations and also illustrated well‐known MRI motion‐related effects. Regarding myocardial tagging, minor differences between [9] and our results can be attributed to slight deviations in the phantom and sequence definitions. As for the flow validation experiment, the magnetization profiles obtained under different flow velocities showed close agreement with those from Reference [23]. Quantitatively, the analysis confirmed that the numerical error decreases with smaller time steps, reaching values below 1%. Our replications of the phase contrast experiments originally conducted with CMRsim [6] and JEMRIS [12] exhibited strong qualitative and quantitative agreement. The small deviation in the boxplot of our simulation and the ground truth was not statistically significant and could be made smaller with a more careful design of the velocity encoding gradients. As for JEMRIS, differences in retrieved velocity can be attributed to the approximated noise addition, the parameters of which were not detailed in Reference [12]. Nevertheless, the reported deviation of ± 10 mm/s in Reference [12] supports the consistency of our implementation. In terms of simulation times, our solution outperforms MRISIMUL [9], although not directly comparable since their result was reported in 2014. Unlike MRISIMUL [8, 9] and its successors [10, 29], our implementation is open‐source. Compared to CMRsim, our method is approximately five times faster when excluding trajectory computation and maintains a slight performance advantage (∼10%) when including it. This speedup is mainly due to (1) pre‐computation of spin trajectories^9^ rather than dynamic seeding, and (2) Julia‐based implementation outperforms Python‐based CMRsim. The speedup over JEMRIS is even more substantial: While JEMRIS requires 330 min on 600 CPU cores [12], our solution achieves approximately 380× speedup (Table S3h) on an 8‐core *desktop* computer. This advantage results from three factors that distinguish KomaMRI from JEMRIS:

1. the *sequence‐aware time‐stepping* [1],
2. native GPU parallelization [1], and
3. recent GPU kernel optimizations,^10^ which provide an additional ∼5× speedup.

The overall speedup is slightly reduced in simulations that involve motion, compared to static cases, since the functions responsible for updating spin positions, although already executed on the GPU, are not yet fully optimized. Our future work includes improving this part of the pipeline.

One advantage of our phantom model is its ability to accommodate arbitrary motion within Path or FlowPath. These can be populated using external software or directly from Julia. In our case, trajectories were generated using different sources: VTK‐m for the aorta and U‐bend, analytical functions for the myocardial tagging and the TOF experiments, and an external dataset for the JEMRIS comparison. The TimeCurve then provides an absolute time ground to execute the motion or to control its periodicity. In addition to non‐rigid motion or flow, arbitrary trajectories can also model diffusion. Original diffusion in KomaMRI was based on a random walk model^11^ and now includes the sampling of an arbitrary ensemble average propagator (EAP) [30]. For spin trajectory computation, accuracy depends on the time step of the solver, which determines both the interval for updating particle positions and the spins that leave the phantom volume, to trigger re‐seeding when needed. This parameter is fully configurable and plays a central role in balancing accuracy and computational cost: Smaller steps yield higher precision but also increase file size and preprocessing time, albeit without additional overhead when trajectories are reused in several simulations. For the comparison with CMRsim, we selected a time step that ensured sufficient accuracy while still achieving faster computation times. Additionally, a high spin density is required to produce reliable results. This requirement is independent of whether trajectories are pre‐calculated or obtained on‐the‐fly and is particularly relevant for reproducing spoiling effects under flow motion conditions [12]. Non‐spoiled steady‐state sequences are generally less demanding in this regard. Overall, there exists a trade‐off between accuracy and computational efficiency, with the main complexity residing in phantom generation rather than in the MRI simulation itself. The optimal balance depends on the specific application, and we are currently investigating both solver time step and spin density to determine appropriate values for different scenarios. Our design provides versatility and efficiency, decoupling trajectory generation from the simulation itself: Motion can be computed once (with an appropriate sampling pattern for the trajectory) and reused in multiple simulations (with a sequence‐aware time stepping for each). While this approach increases storage and memory consumption—potentially reaching several gigabytes—the required disk space is generally manageable, given the capacities of modern computers. RAM may be an issue for phantom loading. Although we have been able to load most of our phantoms in an 8 GB laptop, this amount of RAM did not suffice for the non‐turbulent flow in the U‐bend experiment (Figure 9b); so we recommend at least 16 GB. For GPU execution, only a portion of each trajectory is needed at a time, since simulations are divided into temporal blocks, the number of which can be adjusted. Beyond this, phantom data can be partitioned into smaller chunks that fit within GPU memory constraints, and which are sequentially sent to the GPU and processed. While this process is not yet automated by the simulator, manual partitioning is straightforward in Julia and detailed in the KomaMRI documentation.^12^ Following this approach, we have run our flow experiments in a 4 GB GPU. We maintain a constant number of spins throughout the simulation. This allows for moderate spin counts, avoids tracking non‐contributing spins, and enables the use of regular matrices for efficient computations. Magnetization reset for spins entering the volume should be considered carefully; phantom design should separate inflow regions from those affected by RF pulses to prevent unintended excitation. Non‐rigid motion and flow can also lead to local spin accumulation or depletion, affecting signal intensity [31]. A potential solution is to modulate proton density based on local deformation, as proposed for diffusion simulations [32]. A limitation of the current implementation is that a single TimeCurve is shared by all spins, which poses an efficiency loss when these present different dynamics. This was reflected in the need to store 400 positions per spin in the JEMRIS and CMRsim comparisons. Fortunately, the solution is conceptually straightforward. Additionally, due to this large amount of information to handle, computation times increase considerably compared to static simulations, with the increase being directly related to the complexity of the motion. This can be alleviated by optimizing the internal functions in get_spin_coords and using kernel programming with KernelAbstractions.jl and AcceleratedKernels.jl. Finally, another limitation is the lack of non‐uniform or on‐the‐fly trajectory computation, since our VTK‐m solver currently only supports pre‐computed trajectories with uniform time steps. We are working to extend the solver to incorporate these functionalities, so that users may flexibly choose among (1) equidistant pre‐computation, (2) sequence‐adapted pre‐computation, or (3) fully on‐the‐fly trajectory generation, depending on their needs. With respect to our proposal of file format for phantom exchange, we aim to set the grounds for creating repositories of phantoms directly usable in all format‐compliant simulators. The adoption of the HDF5 standard facilitates interoperability by allowing developers to easily implement the format within their own simulation tools, while also simplifying the I/O processes associated with these data.

## 5 Conclusions

In this paper, we presented a novel dynamic phantom model for the simulation of both simple and complex motions with KomaMRI. The model supports arbitrary spin trajectories and makes use of the concept of TimeCurve for fine‐tuning the temporal behavior of the actions. The experiments have shown very good agreement with other reported solutions, and with much lower computing times. Additionally, we defined a new file format (.phantom) for digital phantom storage and exchange, laying the groundwork for the adoption of a common standard across different MRI simulation platforms.

## Supporting information


**CODE S1** Comparison of Phantom structure versions. (a) Original (KomaMRIv0.8) Phantom structure. (b) Proposed Phantom structure (KomaMRIv0.9).

**FILE S2** Phantom file format specification, which is also available at: https://juliahealth.org/KomaMRI.jl/phantom‐format.

**TABLES S3** Detailed settings and computation times for the experiments described in Sections 2.4 and 3.

## Data Availability

KomaMRI is open‐source, and its code—including our contribution described in Section 2.2—is available on GitHub: https://github.com/JuliaHealth/KomaMRI.jl. The official KomaMRI documentation provides comprehensive explanations of the API functions, the phantom file format, and usage examples: https://juliahealth.org/KomaMRI.jl. All the experiments described in Sections 2.4 and 3 are available in a dedicated GitHub repository: https://github.com/pvillacorta/KomaMotionExperiments/tree/main/mrm. The corresponding phantom files are automatically downloaded when running the experiments, but they can also be accessed independently from Zenodo: https://zenodo.org/records/15591102. Last, the VTK‐m particle trajectory solver is also open‐source and can be found at: https://github.com/jsierra‐pallares/spinAdvection.

## ENDNOTES

^1^

https://github.com/JuliaHealth/KomaMRI.jl/issues/150.

^2^

https://github.com/JuliaHealth/KomaMRI.jl/issues/62.

^3^
As shown in detail in Supporting Information Code S1b.

^4^
See https://juliahealth.org/KomaMRI.jl/stable/reference/2‐koma‐base/#Motion‐related‐functions for details.

^5^
The particle solver repository link can be found in the “Data Availability Statement” section of this paper.

^6^

https://juliahealth.org/KomaMRI.jl/realistic‐head‐motion/.

^7^
Available in https://www.vascularmodel.com/.

^8^
CMRsim repository: https://gitlab.ethz.ch/ibt‐cmr/mri_simulation/cmrsim.

^9^
The phantom files used are available on Zenodo and already contain the precomputed spin trajectories. Hence, this preprocessing step does not need to be repeated to reproduce the results.

^10^

https://juliahealth.org/KomaMRI.jl/stable/explanation/4‐gpu‐explanation/.

^11^

https://juliahealth.org/KomaMRI.jl/stable/tutorial/06‐DiffusionMotion/.

^12^

https://juliahealth.org/KomaMRI.jl/stable/how‐to/4‐run‐distributed‐simulations.
