# Supplementary material for: Versatile and Highly Efficient MRI Simulation of Arbitrary Motion in KomaMRI
Source: Magn Reson Med. 2025 Oct 27;95(3):1791–803. doi: 10.1002/mrm.70145 (PMC12746406; doi:10.1002/mrm.70145)
Supplement: Supplementary file 1 — CODE S1 Comparison of Phantom structure versions. (a) Original (KomaMRIv0.8) Phantom structure. (b) Proposed Phantom structure (KomaMRIv0.9). [file MRM-95-1791-s002.pdf]

S1. Comparison of `Phantom` structure versions

October 7, 2025

```
@with_kw mutable struct Phantom{T<:Real}
    name::String = "spins"
    x   ::AbstractVector{T}
    y   ::AbstractVector{T} = zeros(size(x))
    z   ::AbstractVector{T} = zeros(size(x))
    ρ   ::AbstractVector{T} = ones(size(x))
    T1  ::AbstractVector{T} = ones(size(x)) * 1_000_000
    T2  ::AbstractVector{T} = ones(size(x)) * 1_000_000
    T2s ::AbstractVector{T} = ones(size(x)) * 1_000_000
    #Off-resonance related
    Δw  ::AbstractVector{T} = zeros(size(x))
    #Diffusion
    Dλ1 ::AbstractVector{T} = zeros(size(x))
    Dλ2 ::AbstractVector{T} = zeros(size(x))
    Dθ  ::AbstractVector{T} = zeros(size(x))
    #Motion
    ux  ::Function = (x,y,z,t)->0
    uy  ::Function = (x,y,z,t)->0
    uz  ::Function = (x,y,z,t)->0
end
```

(a)

```
@with_kw mutable struct Phantom{T<:Real}
    name::String = "spins"
    x   ::AbstractVector{T}
    y   ::AbstractVector{T} = zeros(size(x))
    z   ::AbstractVector{T} = zeros(size(x))
    ρ   ::AbstractVector{T} = ones(size(x))
    T1  ::AbstractVector{T} = ones(size(x)) * 1_000_000
    T2  ::AbstractVector{T} = ones(size(x)) * 1_000_000
    T2s ::AbstractVector{T} = ones(size(x)) * 1_000_000
    #Off-resonance related
    Δw  ::AbstractVector{T} = zeros(size(x))
    #Diffusion
    Dλ1 ::AbstractVector{T} = zeros(size(x))
    Dλ2 ::AbstractVector{T} = zeros(size(x))
    Dθ  ::AbstractVector{T} = zeros(size(x))
    #Motion
    motion::Union{NoMotion,Motion{T},MotionList{T}} =
         NoMotion()
end
```

(b)

Code S1: Comparison of `Phantom` structure versions. (a) Original (KomaMRIv0.8) `Phantom` structure. (b) Proposed `Phantom` structure (KomaMRIv0.9).
