# Supplementary material for: Versatile and Highly Efficient MRI Simulation of Arbitrary Motion in KomaMRI
Source: Magn Reson Med. 2025 Oct 27;95(3):1791–803. doi: 10.1002/mrm.70145 (PMC12746406; doi:10.1002/mrm.70145)
Supplement: Supplementary file 2 — FILE S2 Phantom file format specification, which is also available at: https://juliahealth.org/KomaMRI.jl/phantom‐format. [file MRM-95-1791-s001.pdf]

S2. Definition and Format for the `.phantom` File

Pablo Villacorta-Aylagas, Carlos Castillo-Passi, Ryan Kierulf,
Rosa-María Menchón-Lara, Justino-Rafael Rodríguez-Galván,
Pablo Irarrazaval, José Sierra-Pallares, Carlos Alberola-López

October 7, 2025

Contents

1 Introduction **1**
1.1 Motivation . . . 1
1.2 Objective . . . 1

2 The HDF5 Standard and File Format **2**

3 The `.phantom` File Format **2**

4 File Hierarchical Structure **4**

1 Introduction

This file contains the complete specification and details of the `.phantom` file format, including a brief overview of the HDF5 standard, a description of each field in the proposed format, and a hierarchical diagram illustrating the full file structure.

An up-to-date version of the `.phantom` file format specification is also available at: https://juliahealth.org/KomaMRI.jl/phantom-format, which may reflect changes or extensions to the format over time.

1.1 Motivation

Spin-level MRI simulations require storing a wide variety of information, including the positions of each spin —which may be subject to motion—, relaxation times T1 and T2, off-resonance effects, and other metadata. This data is often large and structurally heterogeneous, requiring a storage solution that is optimized for both performance and integration with simulation tools.

While standardized formats such as Pulseq exist for describing MRI pulse sequences, there is currently no widely accepted format for storing digital phantoms used in MRI simulation. This lack of standardization hinders interoperability and reproducibility across different simulation platforms.

1.2 Objective

We propose a new file format, `.phantom`, designed to store comprehensive digital phantom data for MRI simulations. The format is intended to accommodate a wide range of essential information, including initial spin positions, motion trajectories, relaxation properties (T1, T2, PD), off-resonance effects, and relevant metadata. Given the complexity and volume of these data, the format is based on HDF5 (see Sec. 2), a well-established and widely supported standard across scientific computing environments.

The main objective of the `.phantom` format is to promote interoperability and reproducibility across different MRI simulation environments by providing a standardized, extensible, and efficient solution to encode, store, and share digital phantoms. This format has been already made fully compatible with the KomaMRI simulator, which includes built-in I/O functions for these new `.phantom` files.

2 The HDF5 Standard and File Format

The Hierarchical Data Format (HDF) defines a model for organizing and storing large volumes of heterogeneous data. This model comprises an abstract data model, a storage model (i.e., the file format), and a set of libraries for managing these files and their internal data structures [1].

The HDF5 file format is widely used for storing scientific data. Its data model consists primarily of four elements, as illustrated in Figure 1:

- **File**: a container for an organized collection of objects, which include groups, datasets, and metadata. These objects are arranged as a rooted, directed graph. Every HDF5 file contains at least one object —the root group— and all other objects are either members or descendants of this root group [1].
- **Group**: analogous to a directory in a file system, a group can contain zero or more objects (groups, datasets, or named datatypes). Every object in the file must belong to at least one group. The root group is a special case and cannot be a member of any other group [1].
- **Dataset**: multidimensional (rectangular) array of data elements [1]. It represents the primary storage unit for numerical or textual data within an HDF5 file.
- **Attribute**: Metadata attached to named objects (such as groups or datasets). Each attribute consists of a name and associated data, with a structure similar to that of a dataset. Attributes are stored alongside the object they describe and are typically used to provide descriptive information or configuration parameters [1].

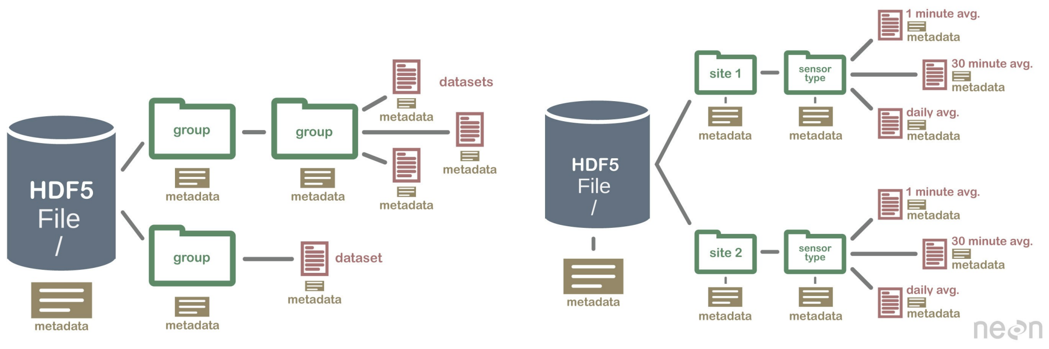

(a) (b)

Figure 1: (a) Diagram of the HDF5 structure. (b) Practical example of use [2].

3 The .phantom File Format

HDF5 has been chosen as the underlying standard for defining the phantom file format. Although these files will carry the `.phantom` extension for identification purposes, their internal structure remains fully compatible with the HDF5 specification.

The proposed format can be divided into four sections: the phantom header information, stored as HDF5 attributes, and three groups containing data related to position, contrast, and motion, respectively.

1. **Header**. General information about the phantom.

```
Version =       # String: identifier of format version
Name =          # String: name of the phantom
Ns =            # Integer: number of spins
Dims =          # Integer <1|2|3>: number of spatial dimensions
```

2. **Position**. Vectors with the initial position of all spins, $\mathbf{x}_n^0$

```
position/x =    # Float: Ns x 1 array with spatial positions in x, always present
position/y =    # Float: Ns x 1 array with spatial positions in y, only if Dims>1
position/z =    # Float: Ns x 1 array with spatial positions in z, only if Dims>2
```

3. **Contrast**. This HDF5 group will contain all the variables affecting the image's contrast: spin density ρ, relaxation constants $T1$ and $T2$, and field deviations or off-resonance $\Delta\omega$. In the future, more parameters can be added.

```
contrast/rho =      # Float: Ns x 1 array with rho values
contrast/T1 =       # Float: Ns x 1 array with T1 values
contrast/T2 =       # Float: Ns x 1 array with T2 values
contrast/T2s =      # Float: Ns x 1 array with T2s values
contrast/Deltaw =   # Float: Ns x 1 array with Deltaw (off-resonance) values
```

4. **Motion**. This group contains all the information necessary for finding the position of each spin at an arbitrary time. As stated in the paper and in the official documentation of KomaMRI [3], KomaMRI represents motion as a collection of elementary entries that can be independently configured and combined. This approach allows for the definition of any complex motion pattern, with the ability to specify overlapping time intervals and even model bidirectional motions along predefined trajectories.

   Therefore, the HDF5 group motion may contain from zero to an arbitrary number of elementary motion subgroups, each named motion_<id>, where <id> is an integer identifier. Each of these subgroups contains three internal groups: action, time, and spins, which define the type of motion, its temporal profile, and the subset of spins it affects, respectively. The possible elements and values that each of these groups may contain are depicted in Section 4. However, for clarity, a brief summary is provided below:

   - action: this HDF5 group contains information about the type and magnitude (i.e., the final state) of the motion. Currently, KomaMRI supports five actions: Translate, Rotate, HeartBeat, Path, and FlowPath. The first three fall under the category of *Simple Actions*, while the last two are considered *Arbitrary Actions*. Simple Actions are defined using scalar parameters, such as translation distances, rotation angles, or contraction rates. Arbitrary Actions, in contrast, are specified through spin trajectories represented as matrices —dx, dy, and dz— with as many rows as involved spins and as many columns as discrete time points stored.
   - time: this group contains the fields of the TimeCurve Julia structure defined in KomaMRI:

     ```
     /motion/motion_<id>/time/type =      # String "TimeCurve"
     /motion/motion_<id>/time/periodic =  # String <"true"|"false">
     /motion/motion_<id>/time/t =         # Float: N_times x 1 array
     /motion/motion_<id>/time/t_unit =    # Float: N_times x 1 array
     /motion/motion_<id>/time/periods =   # Float: N_periods x 1 array
     ```

   - spins: this group specifies which subset of spins in the phantom is affected by the motion. It can be of type AllSpins, meaning that the motion applies to all spins in the phantom, or SpinRange, indicating that only a specific subset is subject to motion. In the latter case, an additional HDF5 attribute named range is included, which stores the affected spin indices as a string-formatted range.

4 File Hierarchical Structure

```
/                                                    HDF5 elements:
├── Version::String                                    Group
├── Name::String                                       Dataset
├── Ns::Int                                            Dimensions
├── Dims::Int                                          Attributes
├── position/ [m]
│   ├── x [Ns x 1]
│   ├── y [Ns x 1]
│   └── z [Ns x 1]
├── contrast/
│   ├── rho    [Ns x 1]
│   ├── T1     [Ns x 1]  [s]
│   ├── T2     [Ns x 1]  [s]
│   └── Deltaw [Ns x 1]  [rad/s]
└── motion/
    ├── motion_<id>/
    │   ├── action/::Action
    │   ├── time/  ::TimeCurve
    │   └── spins/ ::SpinSpan
    │    ...
    └── motion_<id>/
        ├── action/::Action
        ├── time/  ::TimeCurve
        └── spins/ ::SpinSpan
```

TimeCurve

```
...
└── time/
    ├── type::String = "TimeCurve"
    ├── periodic::String = <"true"|"false">
    ├── t [N_times x 1] [s]
    ├── t_unit [N_times x 1] [s]
    └── periods [N_periods x 1] [s]
```

Action types

```
...
└── action/
    ├── type::String = "Translate"
    ├── dx::Number [m]
    ├── dy::Number [m]
    └── dz::Number [m]

...
└── action/
    ├── type::String = "Rotate"
    ├── pitch::Number [º]
    ├── roll::Number  [º]
    └── yaw::Number   [º]

...
└── action/
    ├── type::String = "HeartBeat"
    ├── circumferential_strain::Number
    ├── radial_strain::Number
    └── longitudinal_strain::Number

...
└── action/
    ├── type::String = "Path"
    ├── dx [Ns x N_discrete_times] [m]
    ├── dy [Ns x N_discrete_times] [m]
    └── dz [Ns x N_discrete_times] [m]

...
└── action/
    ├── type::String = "FlowPath"
    ├── dx [Ns x N_discrete_times] [m]
    ├── dy [Ns x N_discrete_times] [m]
    ├── dz [Ns x N_discrete_times] [m]
    └── spin_reset [Ns x N_discrete_times]
```

SpinSpan types

```
...
└── spins/
    ├── type::String = "SpinRange"
    └── range::String

...
└── spins/
    └── type::String = "AllSpins"
```

References

[1] The HDF Group. “HDF5 Documentation.” [Online], Available in: https://support.hdfgroup.org/documentation/hdf5/latest/ (Last accessed: May 15, 2025).

[2] L. A. Wasser. “Hierarchical Data Formats - What is HDF5?” [Online], Available in: https://www.neonscience.org/resources/learning-hub/tutorials/about-hdf5 (Last accessed: Nov. 26, 2024).

[3] C. Castillo-Passi and Contributors. “KomaMRI Documentation.” [Online], Available in: https://juliahealth.org/KomaMRI.jl/stable/ (Last accessed: May 15, 2025).
