# Supplementary material for: Versatile and Highly Efficient MRI Simulation of Arbitrary Motion in KomaMRI
Source: Magn Reson Med. 2025 Oct 27;95(3):1791–803. doi: 10.1002/mrm.70145 (PMC12746406; doi:10.1002/mrm.70145)
Supplement: Supplementary file 3 — TABLES S3 Detailed settings and computation times for the experiments described in Sections 2.4 and 3. [file MRM-95-1791-s003.pdf]

S3. Experimental Settings and Computation Times

October 7, 2025

Hardware Specifications

Hardware specifications

	**Computer name**	
	desktop	***server***
**CPU**	AMD Ryzen 7 5800X 8-Core 3.8 GHz	MD EPYC 7513 32-Core 2.6 GHz
**RAM**	32 GB	1 TB
**GPU**	NVIDIA Quadro RTX 4000 8 GB	4 $\times$ NVIDIA RTX A5000 24 GB

Table S3a: Specifications of the computing systems used to perform the experiments.

1 Illustrative evaluation

Time of flight on a user-defined flow phantom (Fig. 5)

Phantom		
Description	Vertical cylindrical tube with internal flow	
Source	User defined	
Length	40 mm	
Outer radius	10 mm	
Inner radius	4.5 mm	
Flow velocity	4 cm/s	
Duration	Continuous	
Spin distribution	Regular	
Spin separation	0.3 mm	
Number of spins	466,722	
Number of stored positions per spin	500	
`.phantom` file size	1.15 GB	
	**Outer (static) tissue**	**Inner (flow) tissue**
T1	1000 ms	1200 ms
T2	42 ms	92 ms
PD	1	0.9
Sequence		
Description	Cine b-SSFP	
Orientation	Axial, Sagittal	
TR	30 ms	
Flip angle	50º	
RF pulse frequency offset	0	
FOV	40 mm × 40 mm	
Slice thickness	6 mm	
Matrix size	100 × 100	
Number of frames	30	
Simulation		
Computer	*desktop*	
Phantom partition size	250,000 spins	
Computing time	2 orientations × 12 min = 24 min	

Table S3b: Settings for the time-of-flight experiment on a user-defined flow phantom.

Phase contrast on an aorta model (Fig. 6)

Phantom		
Description	Realistic aorta model with flow	
Source	*Vascular Model Repository*<br>(Available in https://www.vascularmodel.com)<br>+<br>VTK-m Trajectory Solver<br>(Available in https://github.com/jsierra-pallares/spinAdvection)	
Dimensions	9.3 cm × 4.1 cm × 7 cm	
VTK-m time step	0.5 ms	
VTK-m trajectory sampling rate	10 ms	
Spin distribution	Random	
Number of spins	2,000,000	
Number of stored positions per spin	100	
`.phantom` file size	3.2 GB	
T1	1400 ms	
T2	200 ms	
PD	1	
Sequence		
Description	Velocity Encoded GRE-EPI multi-shot	
Orientation	Axial, Sagittal	
ETL	16	
TE	16 ms	
TR	60 ms	
V_{ENC} direction	$[x,\ y,\ z]$	
V_{ENC}	50 cm/s	
FOV	110 mm × 110 mm	
Slice thickness	7 mm	
Matrix size	128 × 128	
Total number of acquisitions	2 slices × 3 directions × 2 V_{ENC} = 12	
	**Axial**	**Sagittal**
Flip angle	50º	5º
RF pulse frequency offset	-2kHz	0.8 kHz
Simulation		
Computer	*desktop*	
Computing time (VTK-m)	91 min	
Computing time (MRI simulation)	12 × 2 min 10 s = 26 min	

Table S3c: Settings for the phase contrast experiment on an aorta phantom.

2 Comparative evaluation

Validation of the Bloch solver in the presence of flow (Fig. 7)

Phantom	
Description	1D segment flow phantom
Source	User defined
Length	30 mm
Flow velocity	[0, 80, 120, 160, 200] cm/s
Spin distribution	Regular
Number of spins	400
`.phantom` file size	38 kB
Sequence	
Description	Single slice-selective 90º RF pulse<br>(Fully described by Yuan et al.[4])
Orientation	Axial (flow-transversal)
Slice thickness	7 mm
Slice-selection Gradient amplitude	10 mT/m
RF duration	2.6794 ms
RF amplitude	0.0175 mT
Blackman Window length, L	4500
Grid size, h	$8.9313 \cdot 10^{-4}$ ms
Simulation	
Computer	*desktop*
Computing time	5 velocities $\times$ 15 ms = 75 ms

Table S3d: Settings for the flow validation experiment.

Myocardial tagging on a user-defined phantom (Fig. 4)

Phantom			
Description	Contracting and expanding 3D ring		
Source	User defined		
Length	80 mm		
Outer/Inner radius	50 mm		
Inner radius	25 mm		
Heart rate	75 bpm		
Spin distribution	Regular		
	**Δx**	**Δy**	**Δz**
Spin separation	0.25 mm	0.25 mm	0.25 mm
Number of spins	7,632,792		
Number of stored positions per spin	2		
`.phantom` file size	815 MB		
T1	900 ms		
T2	50 ms		
PD	1		
Sequence			
Description	Cine b-SSFP with tagging (SPAMM)		
Orientation	Axial		
TR	8 ms		
Flip angle	40º		
RF pulse frequency offset	0		
FOV	150 mm × 150 mm		
Slice thickness	7 mm		
Matrix size	128 × 128		
Number of frames	100		
Simulation			
Computer	*server*		
Phantom partition size	1,000,000 spins		
Computing time	1 GPU	6 h	
	2 GPU	3 h	
	3 GPU	2 h 12 min	
	4 GPU	1 h 40 min	

Table S3e: Settings for the myocardial tagging experiment.

Phase contrast comparison with CMRsim (Figs. 8 and 9)

Orientation	**Sagittal**	**Axial**
Phantom		
Description	Stenotic U-bend with turbulent flow	Stenotic U-bend with non-turbulent flow
Source	CMRsim repository (Available in https://gitlab.ethz.ch/ibt-cmr/mri_simulation/cmrsim) + VTK-m Trajectory Solver (Available in https://github.com/jsierra-pallares/spinAdvection))	
Dimensions	3 cm × 11.8 cm × 19.3 cm	
VTK-m time step	0.16 ms	0.5 ms
VTK-m trajectory sampling rate	1.6 ms	2.5 ms
Duration	650 ms	
Spin distribution	Random	
Number of spins	1,500,000	4,000,000
Number of stored positions per spin	408	261
`.phantom` file size	9.8 GB	11.6 GB
T1	300 ms	
T2	100 ms	
PD	1	
Sequence		
Description	Velocity Encoded Spoiled GRE	
TE	5.3 ms	5 ms
TR	8.9 ms	10 ms
Flip angle	15º	15º
V_{ENC} direction	[NoV_{ENC}, z, z, y, y, x, x]	[NoV_{ENC}, z, y, x]
V_{ENC} (cm/s)	[NoV_{ENC}, 550, 50, 250, 50, 100, 50]	[NoV_{ENC}, 550, 100, 100]
FOV	222 mm × 142 mm	70 mm × 140 mm
Slice thickness	5 mm	15 mm
Matrix size	111 × 71	40 × 80
Total number of acquisitions	7 × 2 V_{ENC} = 14	4 × 2 V_{ENC} = 8
Simulation		
Computer	*desktop*	
Computing time (VTK-m)	39 min	42 min
Phantom partition size	200,000 spins	
Computing time (MRI simulation)	14 × 38.6 s = 9 min	8 x 1 min = 8 min

Table S3f: KomaMRI phase contrast experiment settings used for comparison with CMRsim.

Phase contrast comparison with JEMRIS (Fig. 10)

Phantom	
Description	Two parallel tubes with opposite flow directions
Source	Spin trajectories provided by A. Fortin and imported into a KomaMRI phantom
Dimensions	15.6 cm $\times$ 11 cm $\times$ 1.3 cm
Duration	7210 ms
Spin distribution	Regular
Number of spins	2,069,943
Number of stored positions per spin	400
`.phantom` file size	7.4 GB
T1	850 ms
T2	5 ms
PD	1
Sequence	
Description	Velocity Encoded GRE with flow compensation
Orientation	Axial
TE	10 ms
TR	16 ms
V_{ENC} direction	z
V_{ENC}	20 cm/s
FOV	180 $\times$ 130 mm
Slice thickness	2.5 mm
Matrix size	461 $\times$ 333
Total number of acquisitions	2 V_{ENC}
Simulation	
Computer	*desktop*
Phantom partition size	200,000 spins
Computing time	2 $\times$ 21 min = 42 min

Table S3g: KomaMRI phase contrast experiment settings used for comparison with JEMRIS.

	Input	JEMRIS	KomaMRI
Internal tube (mm/s)			
Mean velocity	-44	-45 ± 10	-50.3 ± 0.2
Max velocity	-88	-89 ± 0	-87.7 ± 2.3
External tube (mm/s)			
Mean velocity	44	43 ± 10	49.5 ± 0.2
Max velocity	88	89 ± 0	88.5 ± 3.0
Computation time	–	2 × 134 h	2 × 21 min

Table S3h: Velocity comparison between the simulation input, JEMRIS, and KomaMRI simulations. Variability in the results arises from averaging 100 simulations with added Gaussian noise. Differences between simulators are attributed to approximate noise modelling in our setup, as the original experiment [1] does not provide details on how noise was incorporated. Computation time was (2 V_{ENC}) × 134 hours in JEMRIS and (2 V_{ENC}) × 21 minutes in KomaMRI.
